# Development and validation of a nomogram for predicting early neurological deterioration in patients with moderate traumatic brain injury: a retrospective analysis

**DOI:** 10.3389/fneur.2025.1512125

**Published:** 2025-02-05

**Authors:** Shen Wang, Ruhai Wang, Chao Han, Haicheng Hu, Hongtao Sun

**Affiliations:** ^1^The First School of Clinical Medical, Lanzhou University, Lanzhou, China; ^2^Tianjin Key Laboratory of Neurotrauma Repair, Characteristic Medical Center of People’s Armed Police Forces, Tianjin, China; ^3^Department of Neurosurgery, Fuyang Fifth People’s Hospital, Anhui, China

**Keywords:** moderate traumatic brain injury, early neurological deterioration, nomogram, risk factors, prediction model

## Abstract

**Objective:**

Early neurological deterioration (END) greatly affects prognosis of moderate traumatic brain injury (TBI). This study aimed to develop and validate a nomogram to predict the occurrence of END in patients with moderate TBI.

**Methods:**

A total of 371 patients with moderate TBI were enrolled and divided into the training (*n* = 260) and validation (*n* = 111) groups at a ratio of 7:3. Univariate and multivariate logistic regression analyses were used to identify the significant factors for END, which were used to develop a nomogram. The discrimination of the nomogram was evaluated using area under the receiver operating characteristic curves (AUC), the calibration was evaluated using calibration curves and Hosmer-Lemeshow tests. Decision curve analysis (DCA) was used to evaluate the net benefit of the model for patients.

**Results:**

In the training group, multivariate logistic regression demonstrated that GCS score, epidural hematoma, intracerebral hemorrhage, fibrinogen, and D-dimer were independent risk factors for END in patients with moderate TBI. A nomogram was constructed using the logistic regression prediction model. The AUCs of the nomogram in the training and validation groups were 0.901 and 0.927, respectively. The calibration curves showed that the predicted probability was consistent with the actual situation in both the training and validation sets. DCA curves demonstrated significantly better net benefit with the model. Then a web-based calculator was generated to facilitate clinical application.

**Conclusion:**

The present study developed and validated a model to predict END in patients with moderate TBI. The nomogram that had good discrimination, calibration, and clinical utility can provide clinicians with an effective and accurate tool for evaluating the occurrence of END after moderate TBI.

## 1 Introduction

Traumatic brain injury (TBI) is a common disease with high rates of disability and mortality, which is one of the leading causes of death and disability in adults (1, 2). Moderate TBI is defined by a Glasgow Coma Scale (GCS) score of 9–12 at admission, with reported incidence rates ranging from 4 to 28% (3). Due to its relatively mild condition, little attention has been paid to moderate TBI in clinical practice. Currently, unlike mild or severe TBI, there are no guidelines providing recommendations for the management of moderate TBI (4). Indeed, patients with moderate TBI are in a dangerous state and at high risk of early neurological deterioration (END). Many of them who “talk and die” after trauma belong to this category (5, 6). END is a common complication after TBI, and is characterized by a sudden decline of GCS more than 2 or conversion to surgical treatment within 72 h after admission (7). Previous studies have shown that the prevalence of END in moderate TBI is as high as 33.3% and END serves as a prognostic factor for unfavorable outcomes, potentially resulting in severe disability or death in patients with moderate TBI (8, 9).

Patients at risk of END often require early, high-level neurological monitoring and interventions, such as intracranial pressure monitoring (10, 11). However, the risk factors for the occurrence of END have not been fully elucidated, which makes its early diagnosis and treatment challenging. Therefore, it is essential to explore its mechanism and the related risk factors after TBI. There is urgent clinical demand for an effective model to predict END in patients with moderate TBI. A nomogram is a visualization tool that integrates indicators from different dimensions, to predict the risk of disease occurrence in a graphical and individualized manner (12). Nowadays, there are few studies reporting risk factors or prediction models for END in patients with moderate TBI (13). Thus, the aim of this study was to explore the risk factors associated with END. Finally, we developed and validated a nomogram to assist in predicting the risk of END in moderate TBI patients.

## 2 Methods

### 2.1 Patient population

We retrospectively reviewed the medical records of 371 patients with moderate TBI who were treated at Fuyang Fifth People’s Hospital (Anhui, China) from January, 2018 to April, 2024. Later, the dataset was randomly partitioned into the training (260 patients) and validation (111 patients) groups in a ratio of 7:3. The inclusion criteria were as follows: (1) All patients were older than 18 years old; (2) A GCS score of 9–12 at admission; (3) Clinically diagnosed with TBI by computed tomography (CT); and (4) Admission within 24 h post-injury. Exclusion criteria: (1) History of central nervous system diseases; (2) Patients with multiple trauma; (3) Missing imaging data; (4) Use of antiplatelet or anticoagulant drugs; (5) Other systematic diseases such as renal dysfunction, hepatic dysfunction, malignant tumors, hematologic disorders, and heart diseases; (6) Nursing and pregnant women; (7) Patients who died in the emergency department; and (8) Incomplete clinical data. This study was performed in accordance with the principles of the Declaration of Helsinki, and the protocol was approved by the Ethics Committee of the Fuyang Fifth People’s Hospital.

### 2.2 Data collection

Data collected included gender, age, basic diseases (hypertension and/or diabetes), mechanism of injury (traffic accident, fall, and other), pre-hospital vomiting, first CT scan time after injury (14), GCS score at admission, post-traumatic epilepsy, length of stay, head CT scan findings and laboratory examinations.

Head CT scan findings. Of the CT scan findings, type of TBI was first recorded as epidural hematoma (EDH), subdural hematoma (SDH), cerebral contusion, traumatic subarachnoid hemorrhage (tSAH), intracerebral hemorrhage (ICH), skull base fracture, and skull fracture.

Laboratory examinations were performed within 30 min of admission. We collected laboratory data including blood platelet count, serum potassium concentration, blood glucose, and serum calcium concentration. Glucose-to-potassium ratio was calculated as serum glucose concentration (mmol/L) divided by serum potassium concentration (mmol/L). For coagulation parameters, thrombin time, prothrombin time, fibrinogen, D-dimer, international normalized ratio and activated partial thromboplastin time were detected.

### 2.3 Definition of END

The END was defined as the occurrence of one or more of the following criteria within 72 h after admission: (a) a decrease in GCS score of 2 points or more from the initial GCS score with no pharmacological sedation; (b) a deterioration in neurological status being sufficient for neurosurgical intervention (7, 15). Accordingly, patients were divided into two groups: the END group and the non-END group.

### 2.4 Outcome assessment

Patient outcomes were evaluated by Glasgow Outcome Scale (GOS) score at discharge. Good outcomes were defined as GOS scores of 4–5, and a score of 1–3 was deemed a poor outcome (16).

### 2.5 Statistical analysis

Statistical analysis was performed using the SPSS 26.0 (SPSS, INC; Chicago, USA) and R software (version 4.0.3).^1^ Categorical variables were presented as numbers with percentages and analyzed with an *x*^2^ test or Fisher’s exact test, whereas continuous variables were expressed as mean ± standard deviation or median (interquartile range) and analyzed using Student’s *t*-test or Mann–Whitney *U* test. Univariate and multivariate logistic regression analyses were conducted to determine the risk factors for END. The variance inflation factor (VIF) was analyzed in the regression model for the detection of multicollinearity with a VIF value of 5 or more referring to multicollinearity (17). The nomogram was constructed based on multivariable logistic regression results. The performance of the nomogram was assessed by discrimination and calibration. The discriminative ability of the nomogram was evaluated by the area under the receiver operating characteristic curve (AUC), which ranged from 0.5 (no discrimination) to 1 (perfect discrimination). The calibration was evaluated using calibration curves and Hosmer-Lemeshow tests. Both discrimination and calibration were evaluated using bootstrapping methods with 1,000 resamples. Furthermore, decision curve analysis (DCA) was used to evaluate the net benefit of the model for patients. Statistical significance was set at *p* < 0.05.

## 3 Results

### 3.1 Clinical characteristics

A total of 371 patients were included in this study. There were 236 males and 135 females, with an average age of 59.41 ± 14.47 years old, ranging in age from 20 to 91 years. The injury was mainly caused by traffic accident (161, 43.4%), followed by falls (77, 20.8%) and others (133, 35.8%). The admission GCS score was 11.62 ± 0.78. 101 (27.2%) patients had a history of hypertension, and 50 (13.5%) patients had a history of diabetes mellitus. Out of the 260 patients in the training group, 50 (19.2%) were diagnosed with END and the mean age was 61 years. In contrast, 14 (12.6%) out of the 111 patients in the validation group were diagnosed with END and the mean age was 58 years. The clinical characteristics of patients in the training and validation groups are summarized in Table 1.

**Table 1:** Characteristics and comparison between the training group and validation group.

Characteristic	Cohort (*n* = 371)		*p* value
	Training group (*n* = 260)	Validation group (*n* = 111)	
Gender, *n* (%)			0.705
Male	167 (64.2%)	69 (62.2%)	
Female	93 (35.8%)	42 (37.8%)	
Age (years)	61.0 (52.0, 71.0)	58.0 (49.0, 69.0)	0.107
Medical history, *n* (%)			
Hypertension	63 (24.2%)	38 (34.2%)	0.047
Diabetes mellitus	31 (11.9%)	19 (17.1%)	0.180
Mechanism of injury, *n* (%)			0.139
Traffic	110 (42.3%)	51 (45.9%)	
Fall	61 (23.5%)	16 (14.4%)	
Other	89 (34.2%)	44 (39.6%)	
Prehospital vomiting, *n* (%)	174 (66.9%)	79 (71.2%)	0.421
GCS score at admission	12.0 (12.0, 12.0)	12.0 (11.0, 12.0)	0.584
First CT scan time after injury (h)	2.0 (1.2, 2.6)	2.2 (1.2, 3.1)	0.161
Post-traumatic epilepsy, *n* (%)	28 (10.8%)	11 (9.9%)	0.805
Type of injury, *n* (%)			
Epidural hematoma	40 (15.4%)	23 (20.7%)	0.210
Subdural hematoma	196 (75.4%)	79 (71.2%)	0.396
Cerebral contusion	238 (91.5%)	106 (95.5%)	0.179
tSAH	233 (89.6%)	103 (92.8%)	0.338
Intracerebral hemorrhage	39 (15.0%)	13 (11.7%)	0.403
Skull base fracture	68 (26.2%)	35 (31.5%)	0.290
Skull fracture	139 (53.5%)	72 (64.9%)	0.042
Laboratory examination			
Platelet count (10^9^/L)	186.0 (151.3, 233.8)	177.0 (155.0, 217.0)	0.582
Potassium (mmol/L)	3.6 (3.2, 3.9)	3.6 (3.2, 3.9)	0.880
Glucose (mmol/L)	7.8 ± 2.4	7.4 ± 2.2	0.131
Glucose-to-Potassium ratio	2.1 (1.7, 2.7)	2.1 (1.7, 2.5)	0.174
Calcium (mmol/L)	2.2 (2.1, 2.3)	2.2 (2.2, 2.3)	0.093
Thrombin time (s)	17.1 (15.4, 18.8)	17.3 (15.6, 18.4)	0.643
Prothrombin time (s)	12.1 (11.2, 12.9)	11.7 (11.0, 12.6)	0.027
Activated partial thromboplastin time (s)	28.2 (25.9, 31.2)	27.4 (25.0, 30.5)	0.126
International normalized ratio	1.1 (1.0, 1.2)	1.1 (1.0, 1.1)	0.075
Fibrinogen (g/L)	2.7 (2.2, 3.2)	2.9 (2.3, 3.7)	0.023
D-dimer (mg/L)	4.9 (2.0, 11.9)	6.0 (2.0, 16.0)	0.257
END, *n* (%)	50 (19.2%)	14 (12.6%)	0.122
Length of stay (days)	16.0 (12.0, 22.0)	16.0 (12.0, 22.0)	0.987
GOS at discharge	5.0 (4.0, 5.0)	5.0 (4.5, 5.0)	0.483

GCS, Glasgow Coma Scale; CT, computed tomography; tSAH, traumatic subarachnoid hemorrhage; END, early neurological deterioration; GOS, Glasgow Outcome Scale.

Of the 371 patients, END was found in 64 (17.3%) patients. The median time of END in patients was 7 h. After the occurrence of END, all patients underwent surgical treatment, and 19 of them had poor prognosis at discharge. The causes of END in patients with moderate TBI included 21 cases of delayed intracerebral hemorrhage, 12 cases of enlarged epidural hematoma, 16 cases of increased subdural hematoma, 7 cases of aggravated brain edema, 6 cases of increased intracerebral hemorrhage, and 2 cases of acute hydrocephalus. There were no cases of non-END patients with poor prognosis at discharge (Table 2).

**Table 2:** Reasons for END.

Reason for END	Number of patients, *n* (%)	Median time (h)
Delayed intracerebral hemorrhage	21 (32.8%)	7.00
Enlarged epidural hematoma	12 (18.8%)	3.75
Increased subdural hematoma	16 (25.0%)	7.00
Aggravated brain edema	7 (10.9%)	22.50
Increased intracerebral hemorrhage	6 (9.4%)	8.50
Acute hydrocephalus	2 (3.1%)	6.75
Total	64 (100%)	7.00

END, early neurological deterioration.

### 3.2 Univariate and multivariate analyses

In the training group, the data of 260 patients were used to establish the nomogram. Of these patients, 50 (19.2%) were diagnosed with END. As shown in Table 3, the END group showed lower initial GCS and first CT scan time after injury, and higher rates of those with post-traumatic epilepsy, EDH, SDH, and ICH than the non-END group. In terms of laboratory tests, fibrinogen levels were lower in the END group than in the non-END group, whereas blood glucose, glucose-to-potassium ratio, and D-dimer levels were higher than in the non-END group (*p* < 0.05). Between groups, there were no significant differences in gender, age, medical history, mechanism of injury, pre-hospital vomiting, cerebral contusion, tSAH, skull base fracture, skull fracture, platelet count, serum potassium concentration, serum calcium concentration, thrombin time, prothrombin time, activated partial thromboplastin time, and international normalized ratio. Patients with END had a longer length of stay and worse outcomes than those without END.

**Table 3:** Characteristics and comparison between the END group and non-END group in training cohort.

Characteristics	END	Non-END	*p* value
Number of patients	50	210	
Gender, *n* (%)			0.306
Male	29 (58.0%)	138 (65.7%)	
Female	21 (42.0%)	72 (34.3%)	
Age (years)	64.0 (57.3, 69.3)	60.0 (51.0, 71.0)	0.214
Medical history, *n* (%)			
Hypertension	15 (30.0%)	48 (22.9%)	0.289
Diabetes mellitus	10 (20.0%)	21 (10.0%)	0.050
Mechanism of injury, *n* (%)			0.822
Traffic	20 (40.0%)	90 (42.9%)	
Fall	11 (22.0%)	50 (23.8%)	
Other	19 (38.0%)	70 (33.3%)	
Prehospital vomiting, *n* (%)	37 (74.0%)	137 (65.2%)	0.237
GCS score at admission	12.0 (10.0, 12.0)	12.0 (12.0, 12.0)	<0.001*
First CT scan time after injury (h)	1.3 (1.0, 2.0)	2.2 (1.4, 2.8)	<0.001*
Post-traumatic epilepsy, *n* (%)	10 (20.0%)	18 (8.6%)	0.019*
Type of injury, *n* (%)			
Epidural hematoma	15 (30.0%)	25 (11.9%)	0.001*
Subdural hematoma	45 (90.0%)	151 (71.9%)	0.008*
Cerebral contusion	48 (96.0%)	190 (90.5%)	0.268
tSAH	44 (88.0%)	189 (90.0%)	0.677
Intracerebral hemorrhage	17 (34.0%)	22 (10.5%)	<0.001*
Skull base fracture	11 (22.0%)	57 (27.1%)	0.457
Skull fracture	31 (62.0%)	108 (51.4%)	0.178
Laboratory examination			
Platelet count (10^9^/L)	190.0 (139.3, 222.0)	185.0 (152.0, 235.0)	0.367
Potassium (mmol/L)	3.4 ± 0.5	3.6 ± 0.4	0.051
Glucose (mmol/L)	8.6 ± 2.7	7.6 ± 2.3	0.004*
Glucose-to-Potassium ratio	2.6 (2.0, 3.1)	2.1 (1.7, 2.5)	<0.001*
Calcium (mmol/L)	2.2 (2.1, 2.4)	2.2 (2.1, 2.3)	0.484
Thrombin time (s)	17.4 (15.7, 19.0)	16.8 (15.4, 18.8)	0.436
Prothrombin time (s)	12.4 (11.4, 13.6)	12.1 (11.2, 12.8)	0.063
Activated partial thromboplastin time (s)	27.3 (23.7, 31.8)	28.3 (26.1, 30.8)	0.357
International normalized ratio	1.0 (1.0, 1.1)	1.1 (1.0, 1.2)	0.135
Fibrinogen (g/L)	1.9 (1.6, 2.5)	2.8 (2.4, 3.3)	<0.001*
D-dimer (mg/L)	17.4 (5.5, 45.6)	3.8 (1.7, 9.2)	<0.001*
Length of stay (days)	23.0 (14.8, 34.3)	15.0 (12.0, 21.0)	<0.001*
GOS at discharge	4.0 (3.0, 4.0)	5.0 (5.0, 5.0)	<0.001*

END, early neurological deterioration; GCS, Glasgow Coma Scale; CT, computed tomography; tSAH, traumatic subarachnoid hemorrhage; GOS, Glasgow Outcome Scale.

*p < 0.05 was considered significant.

Multicollinearity was not observed between the independent variables studied and END. A multivariate logistic regression analysis revealed that lower GCS score (odds ratio [OR], 0.564; 95% confidence interval [CI], 0.348–0.911; *p* = 0.019), EDH (OR, 0.203; 95% CI, 0.065–0.637; *p* = 0.006), ICH (OR, 0.138; 95% CI, 0.041–0.459; *p* = 0.001), fibrinogen (OR, 0.249; 95% CI, 0.130–0.476; *p* < 0.001), and D-dimer (OR, 1.054; 95% CI, 1.029–1.080; *p* < 0.001) were independent risk factors for END (Table 4).

**Table 4:** Multivariate logistic regression analysis for early neurological deterioration.

Variable	OR	95% CI	VIF	*p* value
GCS score	0.564	0.348–0.911	1.113	0.019
Epidural hematoma	0.203	0.065–0.637	1.065	0.006
Intracerebral hemorrhage	0.138	0.041–0.459	1.117	0.001
Fibrinogen	0.249	0.130–0.476	1.048	<0.001
D-dimer	1.054	1.029–1.080	1.088	<0.001

OR, odds ratio; CI, confidence interval; VIF, variance inflation factor; GCS, Glasgow Coma Scale.

### 3.3 Development and validation of the prediction model

**Figure 1:**
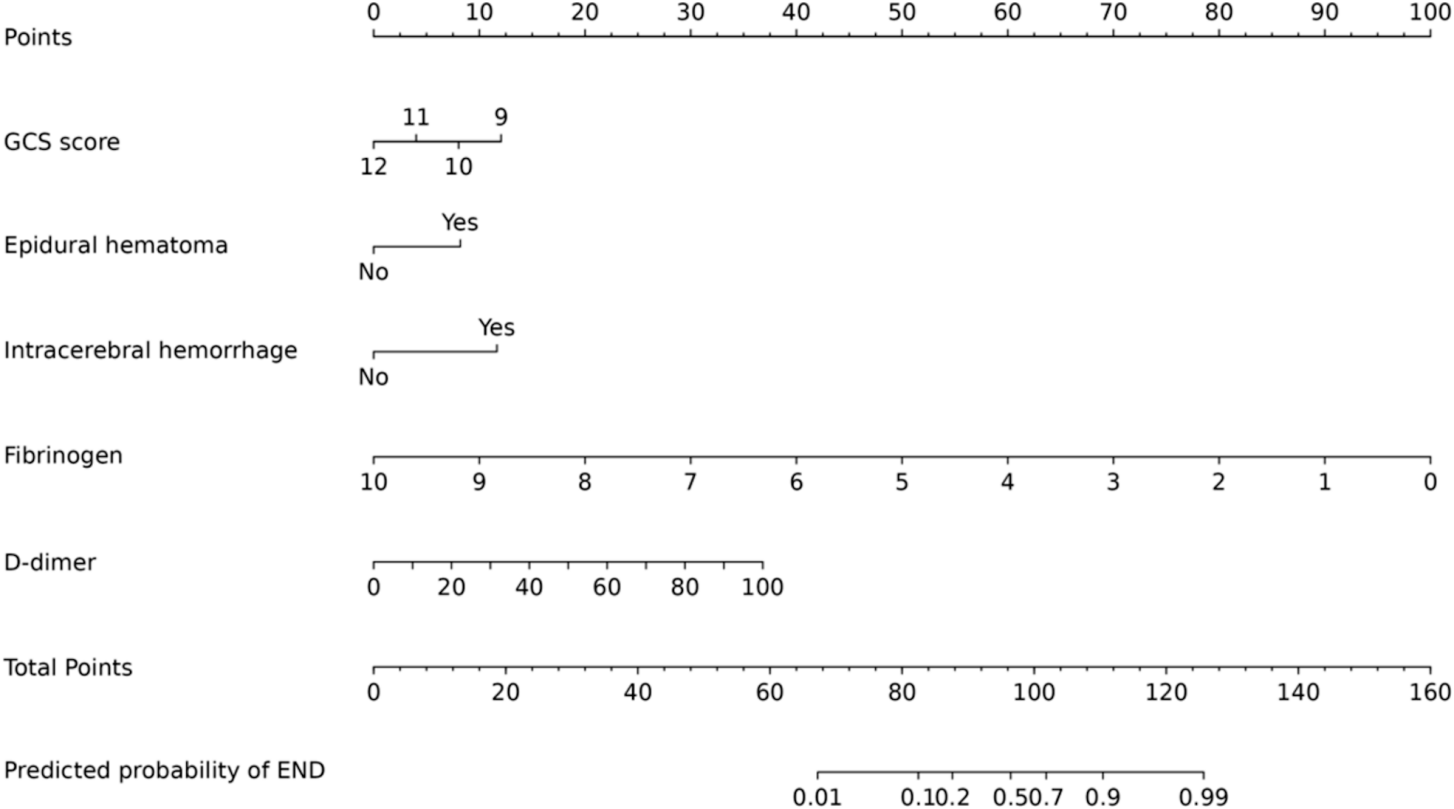
Predictive nomogram model for the risk of early neurological deterioration in patients with moderate traumatic brain injury.

**Figure 2:**
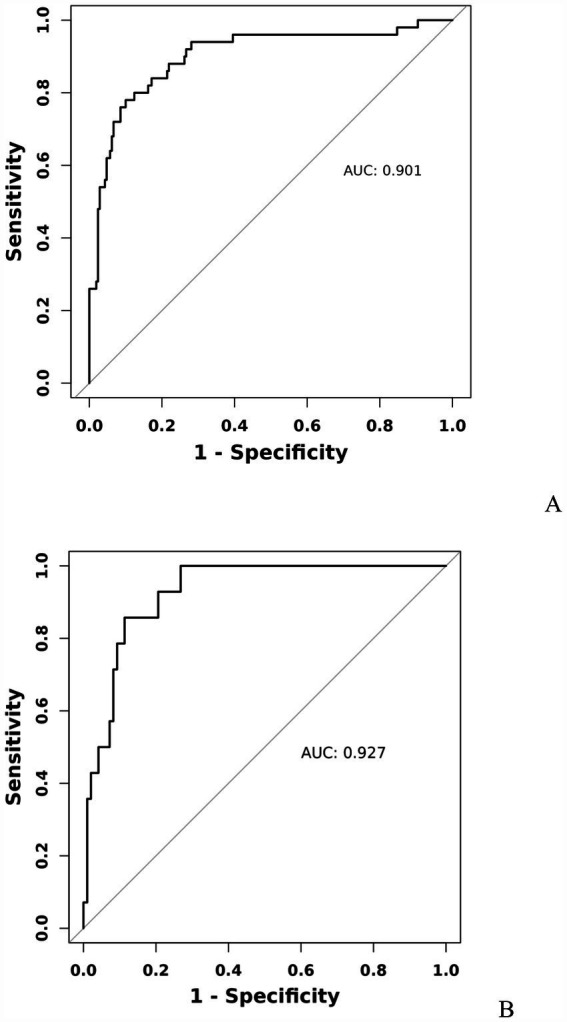
Receiver operating characteristic curve of predicting early neurological deterioration in patients with moderate traumatic brain injury by the nomogram model. Training group **(A)**, Validation group **(B)**.

**Figure 3:**
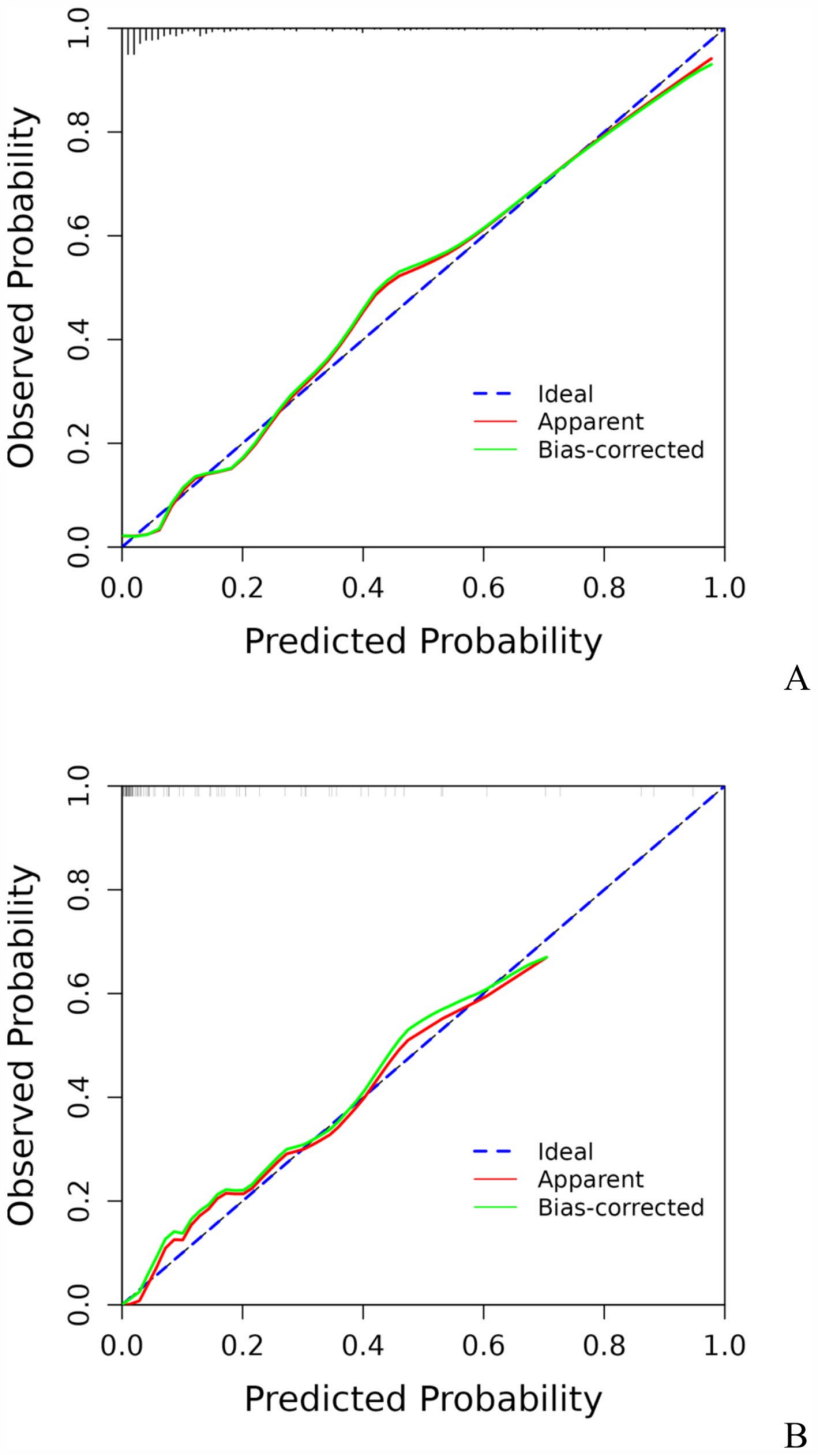
Calibration curve for predicting the risk of early neurological deterioration in patients with moderate traumatic brain injury by the nomogram model. Training group **(A)**, Validation group **(B)**.

**Figure 4:**
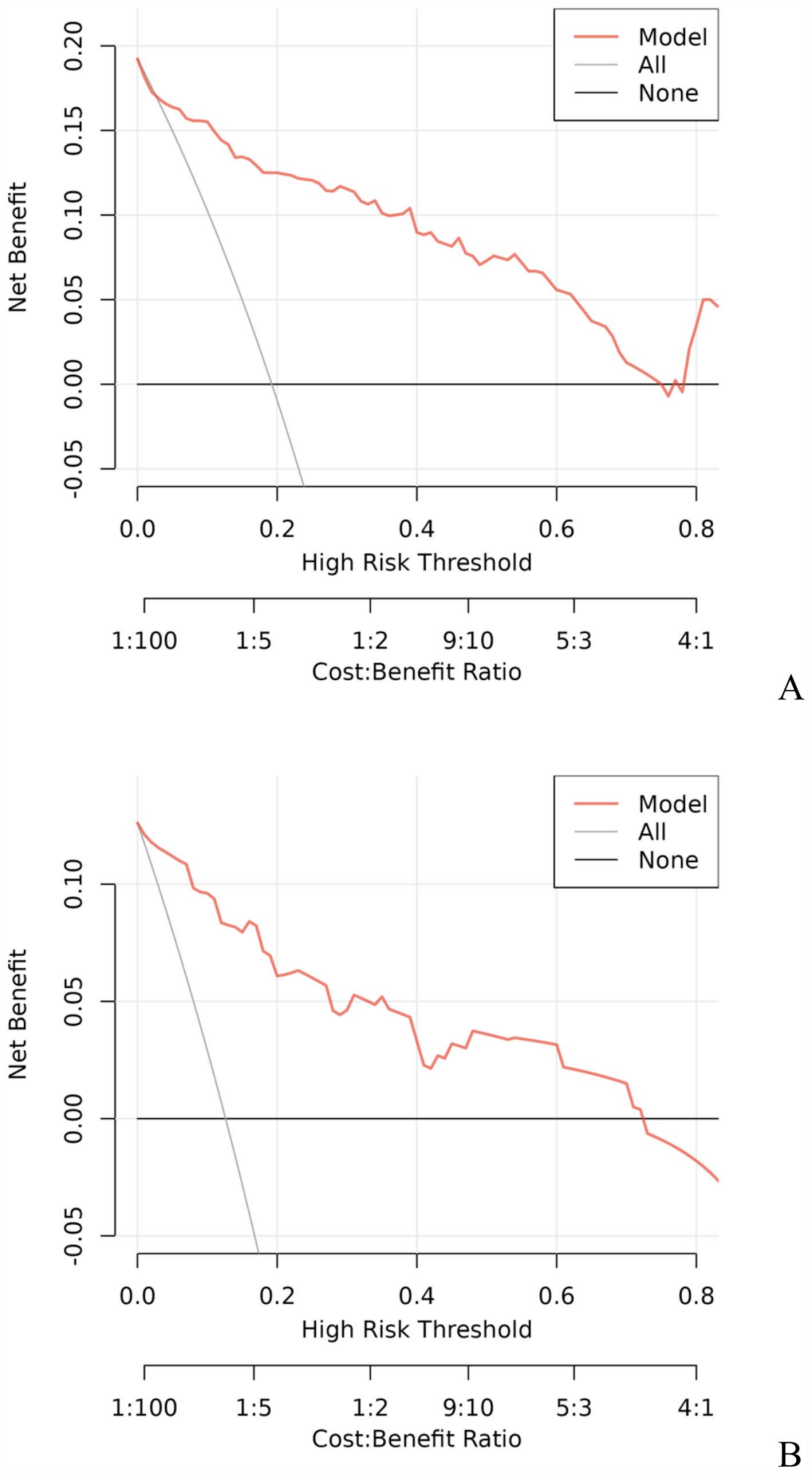
Decision curve analysis for predicting the risk of early neurological deterioration in patients with moderate traumatic brain injury by the nomogram model. Training group **(A)**, Validation group **(B)**.

A model incorporating GCS score, epidural hematoma, intracerebral hemorrhage, fibrinogen, and D-dimer was developed and presented as a nomogram (Figure 1). Each clinical factor corresponds to a specific score, and a straight line is drawn up to the point axis to calculate the total score, which corresponds to the probability of END occurrence. To make this nomogram easier to use, a free online prediction tool is provided here: https://wangshen.shinyapps.io/dynnomapp/. Good discrimination and good calibration were observed in the training and validation sets. The AUC of the nomogram was 0.901 (95% CI, 0.847–0.955) in the training group and 0.927 (95% CI, 0.873–0.981) in the validation group (Figure 2). There was good agreement between the predicted and observed probability in the calibration curves of both groups (Figure 3). In addition, the Hosmer-Lemeshow test produced a nonsignificant *p*-value of 0.173, which can prove the same thing. The DCA curves showed significant net benefit of the predictive model in both the training and validation groups (Figure 4).

## 4 Discussion

Early neurological deterioration was reported in quite a large proportion of patients with moderate TBI, and it is associated mainly with unfavorable outcomes. In order to identify and prevent the occurrence of END as early as possible, it is important and urgent to explore predictors of END. This study found that the five variables collected at admission (GCS score, epidural hematoma, intracerebral hemorrhage, fibrinogen, and D-dimer) were significantly related to END, based on the logistic regression analysis. To our best knowledge, this is the first study to develop a nomogram for evaluating the risk of END in patients with moderate TBI.

The GCS score is the simplest and most reliable method to evaluate the severity of injury in TBI patients. Initial GCS score has been identified as a predictor of poor prognosis in patients with moderate TBI (18, 19). In the present study, GCS score was an independent predictor of END of moderate TBI, and we found that patients with lower GCS scores tended to have a higher risk of END. Nagesh et al. reported that in patients with lower GCS scores, repeat CT scans could exhibit abnormalities during hospitalization (20). Besides, a lower GCS score was found to be associated with an increased risk of contusion expansion (21), which is a major cause of END.

EDH is a collection of blood clots between the outer layer of dura and inner table of skull, which typically occurs from tearing of the middle meningeal artery or dural venous sinuses (22). It is not stable and has a tendency for rebleeding, resulting in sudden changes and deterioration of the patient’s condition, with a life-threatening brain herniation occurring within a few hours. Wang et al. found that EDH is most likely to enlarge in the first 6 h but rarely does so after 24 h (23). It has been reported that up to 10.1% of EDHs still tend to grow within 5–6 h after injury (24). In a study by Xiao et al. it was found that acute EDH without surgical intervention in the early stage may be associated with hematoma enlargement when CT time was added (24). The present study demonstrated that enlarged epidural hematoma was an independent risk factor for END, which was consistent with the previous reports (25). Thus, an aggressive CT repeat strategy should be employed in EDH to assess the progression of hematoma, especially in the early stages after TBI, and these patients often require surgical intervention because of the higher possibility of END during follow-up.

The ICH was also found to be a predictor of END in patients with moderate TBI. TBI is frequently accompanied by intracranial hemorrhage, with progressive ICH being the most common type of progressive hemorrhagic injury (23). The findings of this study indicate that another cause of END is delayed intracerebral hemorrhage, making it necessary for patients with moderate TBI to undergo repeat head CT scans. Studies have shown that ICH can contribute to cerebral edema and increase intracranial pressure (26), additionally, ICH has been proven to be associated with the development of acute hydrocephalus, which can increase intracranial pressure, potentially leading to brain injury, herniation and END (27). In patients with ICH, the volume was greater than 30 mL, and patients were at a high risk of hemorrhagic progression (28), therefore, it is recommended that patients with moderate TBI accompanied by ICH receive immediate surgical treatment to prevent the occurrence of END.

Fibrinogen, a glycoprotein mainly synthesized by the liver, is the major structural component of a clot (29). During the coagulation process, fibrinogen is converted into fibrin by thrombin activation, and participates in platelet aggregation through a cross-linked fibrin network (30). Many studies have revealed that patients with TBI have a significant decrease in fibrinogen levels in the early stages, which is correlated with the severity of brain injury (31). Peng et al. reported that lower fibrinogen levels were predictive for progressive hemorrhagic injury after TBI (32). In another retrospective study involving 106 patients with severe TBI, authors found that fibrinogen at admission was independently associated with the occurrence of progressive hemorrhagic injury and was the main cause of neurological deterioration (33). Moreover, fibrinogen level is also considered to be an independent risk factor for the enlargement of intracranial hemorrhage. One such study reported a negative correlation between plasma fibrinogen levels and the volume of hematoma in TBI patients (34). In the present study, low fibrinogen level was also identified as a predictor of END. Therefore, patients with moderate TBI with lower fibrinogen levels may be at a higher risk of hematoma expansion, which can further explain the association between low fibrinogen levels and END, and poor prognosis.

High D-dimer levels were also demonstrated to predict END. D-dimer is a soluble fibrin degradation product deriving from the plasmin-mediated degradation of cross-linked fibrin, and is one of the most important indicators of coagulation function. Its presence reflects concomitant activation of both coagulation and fibrinolysis (35). Selladurai et al. reported that the incidence of abnormal coagulation following TBI was 40% (36), and patients with high D-dimer levels experienced a greater frequency of unfavorable outcomes after TBI (37, 38). This study demonstrated a significant association between elevated D-dimer levels and the occurrence of END in patients with moderate TBI. Although the mechanism of the impact of D-dimer on END was not well defined, a recent study confirmed that D-dimer was a significant independent predictor of hemorrhage progression (39). Tong et al. stated that an increase in D-dimer correlated with progressive hemorrhagic injury (40). Similarly, Peng et al. suggested that higher D-dimer at admission could predict progressive hemorrhage after head trauma (32). In addition, higher D-dimer levels were found to be associated with a higher risk of contusion progression (41, 42), which is a major cause of END. With regard to logistic regression analysis of multiple factors, D-dimer level was strongly related to the occurrence of END, which indicates that the higher D-dimer level, the greater likelihood of END.

Early detection is a key to preventing the occurrence of END. This is best accomplished by identifying high-risk patients. Consequently, these patients need to be under observation, followed up, and should undergo dynamic-CT review. Early intervention and treatment are needed after END is found. The nomogram is one of the methods to present the predicted model as the graphic scoring. After performing the multivariate regression analysis, we included GCS score, epidural hematoma, intracerebral hemorrhage, fibrinogen and D-dimer, a total of five factors, as nomogram score points. These variables represent the central nervous system function, severity of injury and coagulation status of TBI patients, which basically encompass the degree of anatomical damage and pathophysiological alterations in the body following trauma. Furthermore, the results can be obtained within a relatively short period of time after patient admission, making it convenient, fast, and easy to use. The nomogram prediction model had good discrimination and clinical utility. The AUC for predicting the risk of END occurrence in the training group was 0.901, and also reached 0.927 in the validation group. The calibration plot almost perfectly fit the actual situation. DCA analysis showed that the nomogram exhibited excellent clinical applicability in predicting the risk of END, and the prediction model also performed well in the goodness-of-fit test. The above indicates that the nomogram prediction model may play a positive role in predicting the risk of END in patients with moderate TBI. Compared with traditional clinical prediction methods, the nomogram model can more intuitively show the relationship between various risk factors and prognosis, providing a quantitative prediction tool for clinicians (43). In addition, the model helps to improve the accuracy of prediction and provides the basis for patients to develop individual treatment plans. In patients with moderate TBI, targeted treatment measures can be formulated according to the relevant risk factors to adjust the treatment regimen, thereby effectively improving the prognosis of the patient.

This study has several limitations. First, this was a retrospective study and may therefore have a certain degree of selection bias. Second, a limited number of patients were recruited from a single institution. The prediction model was only subjected to internal validation, lacking external validation, it is necessary to conduct large sample and multicenter studies to prove the feasibility of the nomogram and increase the possibility of extensive popularization of the model. Third, blood parameters were recorded only once on admission and were not monitored dynamically. Last, our model only focuses on the END of patients with moderate TBI, which, although important for clinical practice, does not assess long-term prognosis. To overcome these limitations and further improve the predictive accuracy of the model, we plan to adopt a multi-center, multi-regional approach in future studies to collect more diverse patient demographic data. This will help us build a more comprehensive and accurate model to better predict the risk of END or prognosis of moderate TBI patients.

## 5 Conclusion

In this study, we found that GCS score, epidural hematoma, intracerebral hemorrhage, fibrinogen, and D-dimer were predictors of END in patients with moderate TBI. A prediction nomogram for assessing the risk of END was developed, and the receiver operating characteristic curve, calibration plot, and DCA were used to demonstrate that the nomogram had a good predictive performance, calibration, and clinical utility. It might be a reliable and easy-to-use tool to predict END in moderate TBI patients.

## Data Availability

The raw data supporting the conclusions of this article will be made available by the authors, without undue reservation.

## Footnotes

^1^ https://www.Rproject.org
